# The Controversial Relationship between Body Mass Index and Handgrip Strength in the Elderly: An Overview

**DOI:** 10.21315/mjms2023.30.3.6

**Published:** 2023-06-27

**Authors:** Nadine Soraya, Edy Parwanto

**Affiliations:** 1 Medical Science Study Program, Faculty of Medicine, Universitas Trisakti, Indonesia; 2 Department of Biology, Faculty of Medicine, Universitas Trisakti, Indonesia

**Keywords:** elderly, body mass index, handgrip strength, muscle strength

## Abstract

Body mass index (BMI) is one of the most commonly used anthropometric measurements. BMI is measured by dividing an individual’s weight by their height. In the elderly, the aging process causes physiological changes to organ systems as well as body composition. The most noticeable changes occur in the musculoskeletal system—that is, of decreased muscle strength. Handgrip strength is one of the most commonly considered criteria to measure muscle strength. Various factors, including age, gender and anthropometric measurements such as BMI, are known to affect a person’s muscle strength. In addition, weight and height affect the handgrip strength of an elderly person. However, whether BMI directly affects handgrip strength in the elderly is still debated. Several studies have stated that BMI has a relationship with handgrip strength in the elderly, while other studies have found no relationship between BMI and handgrip strength. BMI and handgrip strength is still controversial and requires further research.

## Introduction

The term elderly is used to refer to people aged 60 years old or over (1). The World Health Organization (WHO) has predicted that the world’s elderly population will double by the year 2050 and that the population over the age of 80 years old will reach around 400 million (2). The aging process causes a person’s physiological function to decrease (3). The changes that take place in the elderly include changes in the musculoskeletal system, which causes a decrease in muscle strength (4). Generally, muscle strength is determined by using a handgrip dynamometer to measure the strength of one’s hand muscles (5). Handgrip strength is a commonly used estimate of upper extremity muscle strength and a good indicator of a person’s nutritional status (6). It is physiologically influenced by many factors, including age, gender, height, weight and body mass index (BMI) (7).

Muscle strength refers to a muscle group’s ability to work to withstand a given load (8). Decreased skeletal muscle health is associated with modifiable factors, including physical activity, nutrition, anthropometry, BMI, bone mineral density and vitamin D status. Malnutrition is also a risk factor for decreased muscle mass, muscle strength and physical function (9). A decrease in handgrip strength can decrease a person’s ability to perform daily activities and thus impact quality of life (10). Moreover, it has been proven that the greater the age and BMI of an elderly person, irrespective of gender, the greater the impact on muscle strength (11).

BMI is defined as a person’s weight in kilograms (kg) divided by height in meters squared (m^2^) (12). BMI determines a person’s anthropometric status (13). There are several classifications of BMI, such as those determined by the WHO and WHO Asia-Pacific (14) and the national classifications given by the Ministry of Health of the Republic of Indonesia (12). According to the WHO classification, there are four BMI groups: i) underweight; ii) normal; iii) overweight and iv) obese (14). The Ministry of Health of the Republic of Indonesia has classified BMI into three groups: i) thin; ii) normal and iv) fat. The thin group is further divided into two subgroups: heavy skinny and light skinny. Persons classified as heavy skinny have BMIs < 17.0 kg/m^2^, while those who are light skinny have BMIs between 17.0 and 18.4 kg/m^2^. Persons in the normal group have BMIs between 18.5 kg/m^2^and 25.0 kg/m^2^. Persons in the light fat group have BMIs between 25.1 kg/m^2^ and 27.0 kg/m^2^, while those in the heavy fat group have BMIs > 27.0 kg/m^2^ (12).

Several factors are known to influence an individual’s BMI: age, gender, ethnicity, race and muscle mass (15). Furthermore, genetics, diet, physical activity and sleep patterns have also been shown to influence BMI (16). In addition to age, sex hormone binding globulin (SHBG) and testosterone also affect BMI. Serum SHBG levels are inversely related to body weight; an increase in body weight lowers SHBG levels, thus altering sex hormone levels (17). Research has also shown that the molecule polymorphism of SHBG does not affect the circulating levels of SHBG and testosterone. SHBG molecular polymorphisms are partly due to the D327N mutation, and genetic variations in SHBG due to the D327N mutation were found to not affect the circulating levels of SHBG and testosterone (18). Apart from age, sex and genetic factors, dietary factors play an important role in determining BMI. Research results have proven that a BMI < 18.5 kg/m^2^ is associated with a low intake of macronutrients (protein, fat and carbohydrates) and, in turn, a low chronic energy deficiency status (19). The results of a study involving 250 healthy men aged 31 years old–60 years old showed that total testosterone and SHBG levels were negatively correlated with age (*P* < 0.05) (20). Furthermore, a study involving elderly women (mean age 71.8 years old) found SHBG levels to be inversely related to total lean body mass (*r* = 0.621, *P* = 0.003) and quadriceps muscle volume (*r* = −0.464, *P* = 0.052). The results of this study also revealed a relationship between increasing age and SHBG levels in the circulatory system, which contributed to a decrease in the muscle mass and muscle function of elderly women (21).

Research has showed that physical activity is of all factors that affect the BMI of an elderly person and that regular physical activity has a positive effect on the aging process and BMI (22). It has been shown that hormones have an impact on the health of elderly men. Elderly men have a high risk of developing non-vertebral fractures due to high levels of SHBG combined with low levels of bioavailable testosterone and oestradiol (23). Recent studies have shown that SHBG can be used as a reference or marker of clinical disorders (24). In this regard, postmenopausal women who were given isoflavone supplementation were found to have decreased serum SHBG levels (25) and increased bone mineral density (26).

The problem faced today is that there are many pros and cons about the effect of BMI on handgrip strength. Past studies have shown that BMI is indeed related to the handgrip strength of the elderly (27). However, several other studies have shown that BMI does not have a clear relationship with handgrip strength in the elderly and the general population (7). Considering the above, the authors of this paper have conducted a literature review on the relationship between BMI and handgrip strength in the elderly, which is presented herein. BMI is the most often used indicator and practical measure of handgrip strength (28).

### Muscle Strength

Muscle strength is the ability of a muscle or group of muscles to resist a load during activity. Several factors affect a person’s muscle strength: muscle diameter, number of motor units, tension size and speed when contracting, type of muscle fibre, type of contraction, supply blood, motivation level, nervous stimulation and nutritional status (7). Muscle strength has a close relationship with the neuromuscular system. The greater the nervous system’s ability to activate muscle fibres, the greater the muscle strength (8).

A person that reaches the age of 70 years old experiences a decrease in muscle strength by 35%–45% compared to a young age (6). Changes occur in the body composition of the elderly. A person’s body weight continues to increase until the age of 60 years old, after which the weight starts to decrease. In addition to a decrease in muscle mass, as described above, changes in body weight affect body composition, in turn affecting BMI (29). In the present day, society is seeing an increase in the elderly population (30). This requires special attention to be paid to elderly health services. It is especially necessary to pay attention to several factors that contribute to the decrease in muscle strength among the elderly. The decrease in muscle mass is one of the main factors that causes the decline in muscle strength (4). Decreased muscle mass and muscle strength can interfere with one’s bodily functions and reduce an individual’s ability to perform daily activities (31). Decreased muscle mass and strength affect the physical performance, functional ability, and mobility of the elderly. Research even shows that decreased muscle strength can increase the risk of death (32).

The physical activity and protein intake requirements of the elderly are also important because both affect hand grip strength (33). In general, it has been proven that protein intake affects muscle contraction activity. Muscle contraction begins with an action potential that travels along with the motor nerve fibre to the muscle fibre. At each end of the nerve fibre, a neurotransmitter substance called acetylcholine is secreted, which causes the opening of the acetylcholine-gated channel. The channel allows sodium ions to enter the muscle fibre. This causes an action potential that travels along the muscle fibre, causing the depolarisation of the muscle fibre membrane (34). Depolarisation of the muscle fibre membrane causes the release of calcium ions stored in the sarcoplasmic reticulum. These calcium ions create a force that makes actin and myosin move towards each other. The force between actin and myosin is needed for the contraction process. When the muscle finishes contracting, calcium ions are pumped back through the calcium pump and then stored in the sarcoplasmic reticulum until the next action potential (35).

### Handgrip Strength

Handgrip strength is a commonly used estimate of muscle strength, especially upper extremity muscle strength (6). The aging process is associated with a decrease in physical activity, which also contributes to a decrease in muscle mass and physical strength. Notably, handgrip strength is a predictor of general health, disability, cognitive decline and death (36). Since handgrip strength decreases with age, it tends to be low among the elderly. Furthermore, handgrip strength has been correlated with various anthropometric measurements, such as weight, height and BMI. It has been found that BMI has a positive correlation with handgrip strength, indicating that an increase in BMI increases handgrip strength and vice versa (27). In addition to these anthropometric status measurements, arm length, too, has been found to be associated with handgrip strength (37). Handgrip actions are supported by a group of muscles in the arm (38). The expected value for handgrip strength is > 34 kg for men and > 22 kg for women (39). Low handgrip strength is correlated with a risk of falls, disability, decreased health quality and increased mortality (33). Handgrip strength is also influenced by age, gender and body size (40). The results of a previous study showed that handgrip strength tends to decrease as age increases (41). In addition, physical activity and nutritional status are also influential factors. Low physical activity levels and inadequate protein consumption cause handgrip strength to reduce (33).

### Elderly People Population

The Law of the Republic of Indonesia Number 13 of 1998 regarding the welfare of the elderly states that an elderly person is someone who has reached the age of 60 years old or over (1). The elderly population is increasing worldwide and is expected to triple within 50 years. More specifically, it is expected to increase from 600 million in 2000 to more than 2 billion in 2050. Indonesia ranks in the top five countries worldwide for the largest number of elderly people. In 2010, the elderly population in Indonesia reached 18.1 million people; by 2025, this number will double to 36 million people (32).

### Aging Process

The aging process has been proven to cause fat tissue in the body to increase, thereby accelerating the decline in muscle mass and strength (42). The aging process is unavoidable (43), and various mental, social and physical changes occur during this process. Due to the aging process, a person’s body composition changes and these changes are clearly reflected in the individual’s BMI. Elderly people have BMIs that mostly fall within the normal range, followed by the obese category. In addition to an increase in BMI, the aging process also results in decreased muscle strength, causing functional abilities to decrease (30). Aging is a progressive process characterised by a decline in organ structure and function, which makes it an influential risk factor for diseases and disabilities, especially in developing countries. Decreased muscle strength can be seen in people within the underweight group. This is because people who are underweight have less muscle mass and, thus, decreased muscle strength (44). In the elderly, there is a decrease in the function of organs and tissues so that the damage that occurs due to the aging process is difficult to repair (30). The aging process is also associated with oxidative stress and many clinical conditions (45). As mentioned earlier, aging causes changes in body functions, and these changes can interfere with the independence and functional capacity of an elderly person (44). The elderly particularly face limitations in carrying out work that requires mobility (46).

### Physiological Changes in the Elderly

The aging process affects all systems of the human body, including the musculoskeletal system. Due to aging, changes occur in bones, muscles, joints and cartilage, causing bone volume and mass to decrease. There is no exception to this reduction and it affects both men and women of all races. It takes place because of a lack of balance in the process of bone remodelling (47). In addition, a decrease in bone density and minerals also occurs. In the elderly, physiological changes include changes in muscle strength, size and quality. Research has shown that the aging process is correlated with a significant decrease in muscle strength (48). Moreover, the changes that occur in the musculoskeletal system affect the muscle strength of the elderly. In other words, a person who reaches old age experiences a decrease in muscle strength. It has been shown that the decline in muscle strength begins when a person is 50 years old (32).

A decrease in the number and size of muscle fibres leads to muscle atrophy in the elderly. This change in muscle mass is accompanied by an increase in the amount of fat as well as a change in muscle density, which causes muscle function to decrease (49). Changes that occur in tendons include a decrease in cell density, decreased matrix turnover, increased nonenzymatic crosslinks called glycation end products, decreased fibril diameter and reduced modulus of elasticity (50). In general, the aging process also results in anatomical and functional changes in body organs, including changes in the sensory, gastrointestinal, cardiovascular, respiratory, endocrinology, haematology and musculoskeletal systems (51).

In addition to the abovementioned factors, short-term immobilisation decreases collagen synthesis in the elderly. The aging process reduces the proliferation and regeneration abilities of stem cells (48). In the elderly, structural and functional changes occur in the body even without underlying pathological processes (22).

### Factors that Affect Elderly People’s Handgrip Strength

The handgrip strength of an elderly woman aged 60 years old–69 years old is 21.7 ± 5.5 kg, while that of an elderly man in the same age group is 32.9 ± 8.7 kg. The handgrip strength of an elderly woman aged 70 years old–79 years old is 18.2 ± 5.3 kg, while that of an elderly man is 32.7 ± 7.7 kg. The handgrip strength of elderly woman aged 80 years old or older is 13.9 ± 5.3 kg, while that of an elderly man is 23.7 ± 6.7 kg. Differences can be seen in the handgrip strengths of elderly women and elderly men when considering these age groups (*P* < 0.01) (44). Based on these data, it is clear that age and gender affect handgrip strength in the elderly.

A past study on muscle mass in the elderly was carried out by measuring the appendicular skeletal muscle mass index (ASMI). ASMI is calculated by dividing appendicular skeletal muscle mass by the square of height. The study involved 183 men and 217 women. The results of the study showed that the prevalence of low ASMI was 15.5% among elderly men and 24.9% among elderly women. Furthermore, elderly women were found to have significantly lower ASMI scores than men (*P* < 0.0001). Calf circumference and BMI were positively correlated with ASMI (*P* < 0.0001) in this study, while age was inversely related to ASMI (*P* = 0.0024) (9).

A person’s nutritional status affects their body mass. The results of a past study showed that body mass is directly related to handgrip strength. In detail, the BMIs of elderly men and women aged 50 years old–59 years old were found to be 25.59 ± 2.73 kg/m^2^ and 26.79 ± 4.84 kg/m^2^, respectively. The BMIs of elderly men and women aged 60 years old–69 years was found to be 26.11 ± 2.71 kg/m^2^ and 28.48 ± 3.25 kg/m^2^, respectively. Finally, the BMIs of elderly men and women aged ≥ 70 years old were found to be 30.48 ± 3.25 kg/m^2^ and 27.37 ± 4.04 kg/m^2^, respectively. The Pearson correlation coefficient (*r*-value) for the age group of 50 years old–59 years old was 0.577 for men (*P* = 0.001) and 0.830 for women (*P* < 0.001). For the age group of 60 years old–69 years old, the *r*-value was −0.825 for men (*P* < 0.001) and 0.890 for women (*P* < 0.001). For the age group of 70 years old and over, the *r*-value was −0.709 for men (*P* < 0.001) and 0.719 for women (*P* = 0.004) (52).

A person with a BMI value falling within the overweight or obese category tends to have lower muscle strength than that of a person whose BMI is in the normal category. Low muscle strength is also found in people with low body weight and poor nutritional status (10). A study showed that nutritional status affects body mass and that body mass has a direct correlation to handgrip strength (33). Several studies have shown that BMI is related to handgrip strength (6, 27, 53). A study conducted in Africa with a sample of 923 individuals aged > 50 years old showed that they had lower handgrip strength values than the Western population. This was due to the lower BMIs and heights of people in the traditional African rural population compared to those of people in the Western population. In addition, it was also shown that handgrip strength decreased with age. The risk of death among subjects with high handgrip strengths was lower than that of subjects with low handgrip strengths, with a hazard ratio of 0.94 per kg increase (*P* = 0.002). After adjustment for several variables (including gender, age, height and BMI) only handgrip strength remained the predictor of mortality. It was further shown that handgrip strength is not only influenced by BMI but also by gender, age and height (54).

The results of a past study show that gender affects BMI among the elderly. The BMI of elderly men was found to be 24.5 ± 4.2 kg/m^2^, while that of elderly women was 25.7 ± 5.1 kg/m^2^. Thus, the BMI of elderly men was lower than the BMI of elderly women (*P* = 0.007). Handgrip strength was 28.8 ± 9.2 kg among elderly men and 18.9 ± 6.9 kg among elderly women. Thus, handgrip strength was found to be higher among elderly men than among elderly women (*P* < 0.001). In the study, handgrip strength of both elderly men and elderly women was negatively correlated with age (*P* < 0.001) but positively correlated with BMI (*P* < 0.05) (55).

A previous study involving 138 elderly of 80 years old–89 years old and 19 elderly of 90 years old–99 years old showed that handgrip strength is influenced by age. Tewnty-nine subjects (21%) out of 138 (88%) from the age group of 80 years old–89 years old experienced a decrease in handgrip strength (*P* = 0.001). Eleven subjects (57.9%) out of 19 from the 90-year to 99-year age group experienced a decrease in handgrip strength (*P* = 0.001), while eight subjects (42.1%) had normal handgrip strength levels. The results also showed that handgrip strength decreased among subjects with low BMIs (underweight), normal BMIs (normal weight) and high BMIs (overweight). Thirteen out of 24 elderly subjects with low BMI levels, 15 out of 75 elderly subjects with normal BMI levels and 12 out of 58 elderly subjects with high BMI levels experienced a decrease in handgrip strength (*P* = 0.002). Twelve out of 58 elderly subjects with high BMI levels experienced a decrease in handgrip strength, while the 46 other subjects had normal handgrip strength (*P* = 0.002) (56). A different study found handgrip strength to be associated with BMI (*r* = 0.29, *P* = 0.00), age (*r* = 0.44, *P* = 0.00), body weight (*r* = 0.57, *P* = 0.00) and height (*r* = 0.57, *P* = 0.00). It should be noted that age, height and weight were important determinants of the handgrip evaluation (57). However, other studies have shown that BMI has no relationship with handgrip strength in the elderly. For instance, a study involved 15 elderly subjects with an average age of 74.46 years old, BMI of 18.38 kg/m^2^, right handgrip strength of 12.18 kg and left handgrip strength of 12.66 kg. The results of a correlation test showed no relationship between BMI and handgrip strength (*r*-count = 0.358 < *r*-table = 0.514), leg muscle strength (*r*-count = 0.348 < *r*-table = 0.514), back muscle strength (*r*-count = 0.324 < *r*-table = 0.514) or the relative total strength muscle (*r*-count = 0.209 < *r*-table = 0.514) (7).

A study involving 91 elderly—38 men (41.8%) and 53 women (58.2%)—was conducted. Ten percent of the subjects had BMIs < 18.5 kg/m^2^, 35.6% had BMIs between 18.5 kg/m^2^ and 25 kg/m^2^ and 54.4% had BMIs > 25 kg/m^2^. The results of a linear regression test showed that gender (*r* = 0.475, *P* < 0.001) as well as waist circumference (*r* = 0.561, *P* < 0.001) affected handgrip strength. The results also showed that the right handgrip strength of elderly men was greater than that of elderly women (*t* = 19.5, *P* < 0.001) (6).

The other results showed that in the age group of 50 years old–59 years old, the handgrip strength of men (23.27 ± 3.89 kg) was higher than that of women (19.69 ± 1.91 kg) (*P* < 0.001). Among those aged 60 years old–69 years old, the handgrip strength of men (20.30 ± 2.34 kg) was higher than that of women (16.83 ± 2.93 kg) (*P* < 0.001). In the age group of 70 years old, the handgrip strength of men (15.64 ± 2.73 kg) was not different from that of women (19.69 ± 1.91 kg) (*P* = 0.724). It should be noted that the BMIs of the subjects were 25.59 ± 2.73 kg/m^2^ and 26.79 ± 4.84 kg/m^2^, respectively, among men and women aged 50 years old–59 years old; 26.11 ± 2.71 kg/m^2^ and 28.11 ± 4.73 kg/m^2^, respectively, among men and women aged 60 years old–69 years old; and 30.48 ± 3.25 kg/m^2^ and 27.37 ± 4.04 kg/m^2^, respectively, among men and women aged 70 years old. The correlation between BMI and handgrip among men was denoted by *r* = 0.577 (*P* = 0.001), and that among women was *r* = 0.830 (*P* < 0.001) in the age group of 50 years old–59 years old. The correlation between BMI and handgrip in the age group of 60 years old–69 years old was *r* = 0.825 (*P* < 0.001) among men and *r* = 0.890 (*P* < 0.001) among women. The correlation between BMI and handgrip in the age group of 70 years old was *r* = 0.709 (*P* < 0.001) among men and *r* = 0.719 (*P* < 0.004) among women (52).

Research involving 4,644 women and 3,797 men aged 48 years old–92 years old showed that the average BMI was 26.6 kg/m^2^ (14.4 kg/m^2^–59.6 kg/m^2^) among women and 27.1 kg/m^2^ (16.2 kg/m^2^–52.9 kg/m^2^) among men. Only 49 women (1.1%) and 12 men (0.03%) in the study had BMIs < 18.5kg/m^2^. One hundred and six men (2.8%) and 263 women (5.7%) recorded BMIs of 35 kg/m^2^. Handgrip strength increased with increasing waist circumference in both men and women, although the relationship was weak. Handgrip strength of underweight men and women was lower than that of men and women with normal or overweight BMI. Handgrip strength in very obese women is lower than in women who have a normal weight. Moreover, handgrip strength decreased with increasing waist circumference. For every 10 cm increase in waist circumference, handgrip strength decreased by 3.56 kg in men and 1.00 kg in women. The data indicated that BMI was inversely correlated with handgrip strength among obese people. Handgrip strength increased with increasing BMI. Low BMI was correlated with low muscle strength, while a high waist circumference was correlated with low handgrip strength. Furthermore, fat deposition in muscles was found to be a risk factor for decreased muscle strength (58).

In recent research involving an elderly group in South Korea, a high BMI was correlated with hypertension prevalence. Moreover, in a group of elderly men, low relative handgrip strength was correlated with hypertension (59). Notably, strong handgrip strength was correlated with decreased cognitive function among obese women, but the same association was not found in the case of nonobese women. Therefore, handgrip strength can be used as a marker to measure the cognitive functioning of obese women (60).

The preliminary results of a study in Finland involving 2,021 women and men aged 55 years old showed that obesity was correlated with low handgrip strength (*P* < 0.001). The handgrip strengths of a group of non-obese people who used to be obese were not different from those of a group that was never obese. Furthermore, in the currently obese group of people, those who had been obese for 50 years, 40 years and 30 years had lower handgrip strengths than those of the group that was never obese and the group that was now nonobese (but previously obese) (*P* < 0.001). These data revealed a relationship between obesity and handgrip strength in older adults (42).

In addition, decreased muscle strength is also a health problem with a fairly high prevalence among the elderly (61). A study showed that the prevalence of handgrip strength was 16.5% among elderly men and 20.6% among elderly women (62). In addition, it has been demonstrated that age, decreased functional activity, body weight and wrist circumference have a negative relationship with handgrip strength (63). A comparison of research results on the relationship between BMI and handgrip strength in the elderly is presented in Table 1.

## Conclusion

Elderly people are those who have reached the age of 60 years old or over. In the elderly, the aging process that occurs causes physiological changes in all organs of the body. Changes in the muscles cause a decrease in muscle strength. Changes in body composition also occur, affecting the BMI of the elderly. BMI is an anthropometric measure that involves dividing a person’s weight by their height. Decreased muscle strength can be measured by determining one’s handgrip strength. Several factors have been found to affect handgrip strength, including age, gender and various anthropometric measurements. One of the influential anthropometric measurements is BMI. Weight and height affect a person’s BMI. In addition, changes that occur in the weight and height of an elderly person will affect their handgrip strength. Several studies have shown that a relationship exists between BMI and handgrip strength in the elderly. However, other studies have stated that this relationship is only seen in elderly women. One study found no relationship between BMI and handgrip strength in the elderly. Based on the data from our literature review, we conclude that the relationship between BMI and handgrip strength is still controversial and requires further research.

## Figures and Tables

**Table 1:** Comparison of research results on the relationship between BMI and handgrip strength in the elderly

Research title	Research methods and design	Results	References
Factors that influenced handgrip strength in elderly patients at Panti Wredha Tangtu and Geritarian Polyclinic, Sanglah Center General Hospital–Denpasar, Bali, Indonesia	Analytical observational method with a cross-sectional approach	Elderly women had lower handgrip strengths than men. Bodyweight and waist circumference were positively related to handgrip strength. The thinner an elderly person, the lower their handgrip strength	6
The relationship between BMI and muscle strength in the elderly at Panti Wredha Rindang Asih III, Boja District (Kendal Regency, Central Java Province, Indonesia)	Descriptive correlation	No relationship was found between BMI and handgrip muscle strength in the elderly	7
Association between obesity history and hand grip strength in older adults—exploring the roles of inflammation and insulin resistance as mediating factors	Analytical observational method with a cross-sectional approach	An earlier history of obesity was found to correlate with low handgrip strength in the elderl	42
Handgrip strength and flexibility and their association with anthropometric variables in elderly	Analytical observational method with a cross-sectional approach	BMI and handgrip strength were only correlated in women	44
Handgrip dynamometry in elderly individuals and its relation with BMI	Cross-sectional	Handgrip strength decreased significantly with age. The higher the BMI value, the lower the handgrip strength	52
Handgrip strength, ageing and mortality in rural Africa	Analytical observational method with a longitudinal approach	Handgrip strength was found to depend on age, gender, height, and BMI	54
Predictors of handgrip strength among the free living elderly in rural Pahang, Malaysia	Analytical observational method with a cross-sectional approach	Body weight, height, and BMI had positive correlations with handgrip strength. Handgrip strength was higher among men than among women	55
Factors associated with loss of handgrip strength in long-lived elderly	Quantitative study with a cross-sectional approach	Decreased handgrip strength was more common among women. A significant association was found between decreased handgrip strength and the factors of age and BMI	56
Age and anthropometric traits predict handgrip strength in healthy normals	Correlation study with cross-sectional approach	A significant relationship was found between hand grip strength and the factors of age, height, and weight	57
Cross-sectional associations between different measures of obesity and muscle strength in men and women in a British cohort study	Cross-sectional	Based on BMI, greater body mass was found to correlate with greater handgrip strength	58
